# The Heart in the Tuberculous

**Published:** 1906-04-21

**Authors:** 


### THE HEART IN THE TUBERCULOUS.

Dr. Woods Hutchinson [1] complains that in the consideration of pulmonary tuberculosis attention has been too long and too exclusively focussed upon the lung. Running over the varying phases of opinion with regard to the constitutional peculiarities which lend themselves to the development of tuberculosis, he observes that "the more we study the alleged 'strumous' and 'consumptive' types, the more we are driven to two conclusions: first, that almost any undeveloped, languid, or unhealthy child or young adult, from whatever cause defective, could be fitted under one or other of them; second, that such distinctive features as are present in those who afterward become clearly consumptive are almost invariably the signs of an early stage of tuberculosis." But one general physical characteristic may be considered to be a proven predisposer to the attack of tuberculosis, and that is unusual tallness of stature. In the author's opinion, confirmed it appears by a general consensus of authorities, men upwards of five feet eleven inches in height, and women upwards of five feet seven, are distinctly more subject to the attack of the disease than the average person, and succumb to it more readily when attacked. By way of illustration the author gives some data derived from the statistics of two British regiments. One of these is the regiment of Life Guards, "those great hulking be-ribboned and glittering popinjays," as he unkindly calls its members, the other is the Woolwich corps of the Royal Artillery. These regiments are recruited on precisely the same standards except that of height, the minimum height for the Life Guards being six feet, that for the artillery being five feet four inches. The men are fed and housed in a fashion practically identical, and yet the death-rate from tuberculosis among the Life Guards is rather more than double that among the artillerymen.

The next investigation conducted by the author dealt with the shape of the chest in consumptives. He found as a result of some seven hundred measurements that the consumptive chest is rounder and not flatter than that of health. The real interest of the author's contribution centres, however, round an attempt to prove that an under-sized heart, as compared with the body-bulk, is one of the most important factors in the incidence of tuberculosis. He states that Laennec was strongly of opinion that tuberculosis usually occurred in individuals with small hearts, and many other authorities of less note are quoted in a similar sense. In a study by Bouchard and Balthazard, the size of the heart was estimated in ninety patients suffering from pulmonary tuberculosis. The results showed that consumptives might by this means be divided into two categories: first, those who, not being definitely predisposed, yet contracted the disease from the frequency of their opportunities of infection; and secondly, the definitely predisposed. In the first class the heart was normal in size, in the second class it was much diminished. The ancient doctrine that valvular disease of the heart protects against infection of tuberculosis is quite exploded, and has been replaced by the knowledge that such a lesion as pulmonary stenosis renders its victim quite unusually liable to the ravages of the bacillus of Koch.

The attention of the author was first directed to the condition of the heart in the tuberculous by an experience in comparative pathology gathered during a period of two years spent as post-mortem examiner to the Zoological Gardens in Regent Park. Having failed to trace any zoological affinity between the common victims of tuberculosis, the author turned his attention to the question of food. Here a certain parallelism became apparent; thus while tuberculosis can be found among both animal and vegetable feeders in captivity, it never becomes a common cause of death except among the herbivora or the mixed feeders. For instance, although tuberculosis occurs among the carnivorous Canidæ and Felidæ, it never reaches in them more than a low level of frequency, while, as is well known, among herbivora such as cattle, and mixed feeders like monkeys, it assumes the proportions of a pestilence. But even among the vegetable feeders there appear curious variations in susceptibility to tuberculosis. For instance, cattle and antelopes are very subject to the disease, while the equally pure herbivora—sheep, goats, deer, horses, zebras, and asses—are almost exempt. Searching, therefore, for some other factor in the determination of susceptibility, the author turned to the relative size of the heart in proportion to the body-weight. Along these lines there exists a surprising uniformity of results, and one tending to explain away the apparent inconsistencies previously encountered. As a result of measurement it was found that not one of the carnivora, whether bird or animal, has a heart much less than one hundredth of its body-weight, while among the herbivora, whether bird or mammal, none of those whose heart-weight was in excess of this standard was found to be susceptible, except in a very moderate degree. For instance, the carnivorous eagle, hawk, owl, and vulture, with hearts weighing about $\frac{1}{115}$th of their body-weight, suffer about six times as much from tuberculosis as do the parrots with a heart $\frac{1}{80}$th of the body-weight; while the turkey, fowl, and pheasant, with hearts weighing $\frac{1}{250}$th of the body-weight, suffer no less than twelve times as much as the parrots. Among mammals the carnivora, with large hearts, show (except in the case of the domesticated dog) a very low level of susceptibility; while among the herbivora the line of cleavage is marked by the heart-weight in a most striking degree. Thus the deer with a heart-weight of 1 to 90 is almost absolutely immune, while the antelope in the same group, with a heart-weight of 1 to 200, is exceedingly susceptible; and the cow, with a heart-weight of barely 1 to 250, is notoriously victimised.

The contention that this wasting of the heart is part and parcel of the marasmic condition of the whole body under the conditions of tuberculosis is met by the statement that while the diminution in the size of the consumptive heart amounts to no less than some 30 to 40 per cent. of the normal, the total wasting of the body, even in the most extreme cases, does not exceed 15 or 20 per cent.; while the classical experiments of Liebig have shown that in the case of starving dogs the heart's muscle is, next to the central nervous system, the very last tissue to suffer.

In view of these investigations the author is immensely impressed with the importance of the heart condition in determining the incidence and gravity of pulmonary tuberculosis, so much so, indeed, that he threatens to ride his hobby-horse to death. But there is much of interest in the paper, the conclusions of which are as follows:—

That a weak, under-sized, muscularly deficient heart, indicated by a weak, rapid pulse and a defective first sound, is one of the most constant and significant conditions present in consumption.

That this condition in a considerable percentage of cases precedes the development of tuberculosis. The earlier its appearance in the course of the disease, and the more striking its degree, the graver is the prognosis. That in tuberculosis, as in pneumonia, while the chief seat of toxin-production is the lung, the chief strain falls upon the heart, and death in the majority of cases is due to toxic heart-failure.

That the under-sized and inadequate heart is, like the round chest, due to the persistence of conditions normal at about the period of puberty. That the condition of the heart should be our principal guide in the *diagnosis* (the italics are ours), prognosis, and treatment of consumption; if the pulse is slowing and strengthening, never mind the lung.

That a persistently rapid pulse without other ascertainable cause should always rouse suspicion of incipient tuberculosis.

[1] Med. Rec. N.Y., March 3, 1906.

